# Objective parallel-forms reliability assessment of 3 dimension real time body posture screening tests

**DOI:** 10.1186/1471-2431-14-221

**Published:** 2014-09-04

**Authors:** Ireneusz M Kowalski, Halina Protasiewicz-Fałdowska, Michał Dwornik, Bogusław Pierożyński, Juozas Raistenskis, Wojciech Kiebzak

**Affiliations:** Department of Rehabilitation, Faculty of Medical Science, University of Warmia and Mazury in Olsztyn, Olsztyn, Poland; Department of Osteopathic Medicine and Department of Physiotherapy, Medical College of Podkowa Lesna, Podkowa Lesna, Poland; Department of Chemistry, Faculty of Environmental Management and Agriculture, University of Warmia and Mazury in Olsztyn, Olsztyn, Poland; Department of Rehabilitation, Physical and Sports Medicine, Faculty of Medicine, Vilnius University, Vilnius, Lithuania; Institute of Physiotherapy, Faculty of Health Science on University of Jan Kochanowski, Kielce, Poland

**Keywords:** Postural defects, Spinal deformities, Screening tests, Topography

## Abstract

**Background:**

Screening tests play a significant role in rapid and reliable assessment of normal individual development in the entire population of children and adolescents. Body posture screening tests carried out at schools reveal that 50-60% of children and adolescents demonstrate body posture abnormalities, with 10% of this group at risk for progressive spinal deformities. This necessitates the search for effective and economically feasible forms of screening diagnosis. The aim of this study was to assess the reliability of clinical evaluation of body posture compared to objective assessment with the Zebris CMS-10 system (Zebris Medical GmbH).

**Methods:**

The study enrolled 13-15-year-old pupils attending a junior secondary school (mean age 14.2 years). The study group consisted of 138 participants, including 71 girls and 67 boys, who underwent a clinical evaluation of the body posture and an examination with the Zebris CMS 10 system.

**Results:**

Statistically significant discrepancies between the clinical and objective evaluation were noted with regard to lumbar lordosis in boys (n = 67) and thoracic kyphosis in girls (n = 71). No statistically significant differences in both groups were noted for pelvic rotation and trunk position in the frontal plane.

**Conclusions:**

1. The finding of significant discrepancies between the results of assessment in the sagittal plane obtained in the clinical examination and Zebris CMS-10-based assessment suggests that clinical evaluation should be used to provide a general estimation of accentuation or reduction of spinal curvatures in the sagittal plane.

2. The clinical evaluation of posture is reliable with regard to assessment in the frontal plane.

3. The Zebris CMS-10 system makes the clinical examination significantly more objective with regard to assessment of the physiological curvatures and may be used to make screening tests more objective with regard to detecting postural defects.

**Electronic supplementary material:**

The online version of this article (doi:10.1186/1471-2431-14-221) contains supplementary material, which is available to authorized users.

## Background

Human body posture is a motor habit associated with daily activity with an underlying morphological and functional basis [1]. It reflects the psychophysical status of the individual and is an index of mechanical efficiency of the kinaesthetic sense, muscle balance and musculomotor co-ordination [2]. Normal human posture in the vertical position relies on the spine and its position against the head and pelvis [3, 4]. The spatial relations among and between bony structures and articulations are stabilised by a system of fasciae, ligaments and muscles, while the central nervous system is the superior controller of body posture [5, 6]. Body posture variability depends on age, sex and environmental factors influencing its development during body growth [7, 8]. The following conditions are regarded as postural defects: abnormal shape of the physiological spinal curvatures, asymmetrical positioning of the shoulder or pelvic girdle, disturbance of the knee joint axis and abnormal shape of the foot arches. Screening studies of postural defects carried out at schools reveal that 50-60% of children and adolescents demonstrate body posture abnormalities, with 10% of this group at risk for scoliosis or other progressive spinal deformities [9–12]. An alarmingly high percentage of these defects are attributable to poor motor activity of children and adolescents, rapid changes taking place in the body during individual development and excessive time spent in the seated position [13]. An early and reliable detection programme for the population of children and adolescents combined with prophylactic measures to prevent the persistent spinal and trunk deformities is an appropriate strategy that can also mininise the medical and financial outcome of the more complex process of future treatment of postural defects and scolioses that might be necessary. The findings of a clinical evaluation of body posture and trunk asymmetry in a child depend on the experience of the examiner, compliance of the child and availability of bedside diagnostic equipment. Screening tests rely mainly on clinical evaluation since screening is supposed to be available to the entire population of children and adolescents. The easy availability and simple procedure used also need to guarantee a high reliability of diagnoses of postural defects. Non-invasive methods that will make diagnosis easier and more comprehensive are being sought to ensure more objective measurements. The Zebris CMS-10, a system for assessing body posture in three planes, offers a non-invasive method for evaluating the spatial positioning of selected topographic reference points in the frontal, sagittal and transverse planes, thus supplying objective data to support a clinical evaluation. The Zebris CMS 10 system demonstrates a high degree of test-retest reliability, intertester reliability and intratester reliability [14, 15]. The inclinometer method demonstrates a high degree of intertester reliability and intratester reliability [16, 17]. A variation of up to 1.5° was allowed using this technique. Measurements were repeated several times in each participant until two consecutive attempts by two independent examiners yielded the same angle values (including the admissible variation of 1.5°), thus complying with the principles of intertester and alternate-forms reliability. The aim of this study was to assess the reliability of clinical evaluation of body posture compared to objective assessment with the Zebris CMS-10 system.

## Methods

**Figure 1:**
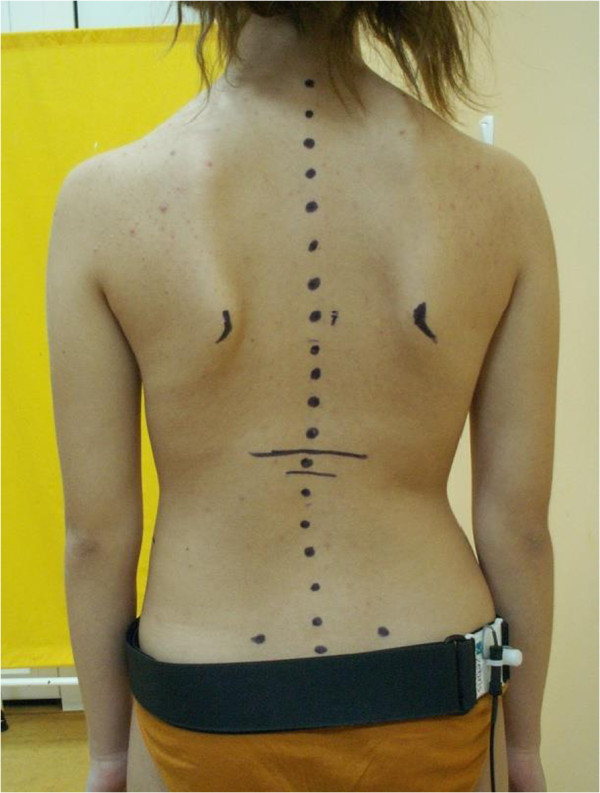
**Marking of anatomical skeletal reference landmarks.**

**Figure 2:**
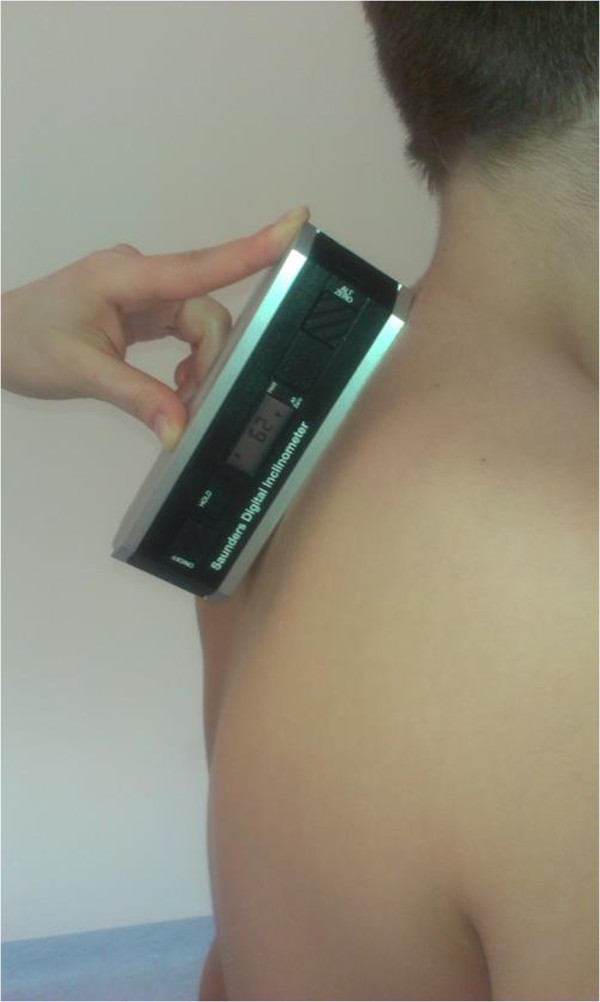
**Clinical examination with Saunders inclinometer.** Reference marker is a belt attached below iliac spines.

The methodology was approved by the Ethics Committee of the Rehabilitation Hospital for Children, Olsztyn, Poland. The study enrolled 13-15-year-old pupils attending a junior secondary school. The mean age was 14.2 (±0.6) years. The study group consisted of 138 participants, including 71 girls (mean age 14.1 ± 0.4 years, mean height 160.3 ± 3.4 cm, mean body weight 64.8 ± 3.9 kg) and 67 boys (mean age 14.4 ± 0.8 years, mean height 166.6 ± 2.9 cm, mean body weight 68.1 ± 3.6 kg). The exclusion criteria were a diagnosis of scoliosis and/or status post spinal surgery and/or feeling any pain. The screening test was carried out with the participants in a free standing position, involving specialists in rehabilitation as examiners and a Zebris CMS 10 system. The objective of the examination was not revealed to the examiners. In the first part, reference skeletal landmarks were marked on the body according to the principles of palpation anatomy. Trunk positioning was evaluated clinically in the sagittal and frontal planes. The findings were recorded in the study protocol (Additional file 1). Thoracic kyphosis and lumbar lordosis were evaluated in the sagittal plane with a Saunders inclinometer. Pelvic rotation was also evaluated in the sagittal plane. A Saunders inclinometer was placed in the cervicothoracic junction with the long arm pointing downwards from the spinous process at the apex of the curve and in the lumbosacral junction with the long arm pointing upwards from the spinous process at the apex of the curve (Figures 1, 2). The respective reference ranges assumed for kyphosis and lordosis were 30-40° and 25-35° [18]. The symmetry of position of the shoulder and pelvic girdles was evaluated in the frontal plane.

**Figure 3:**
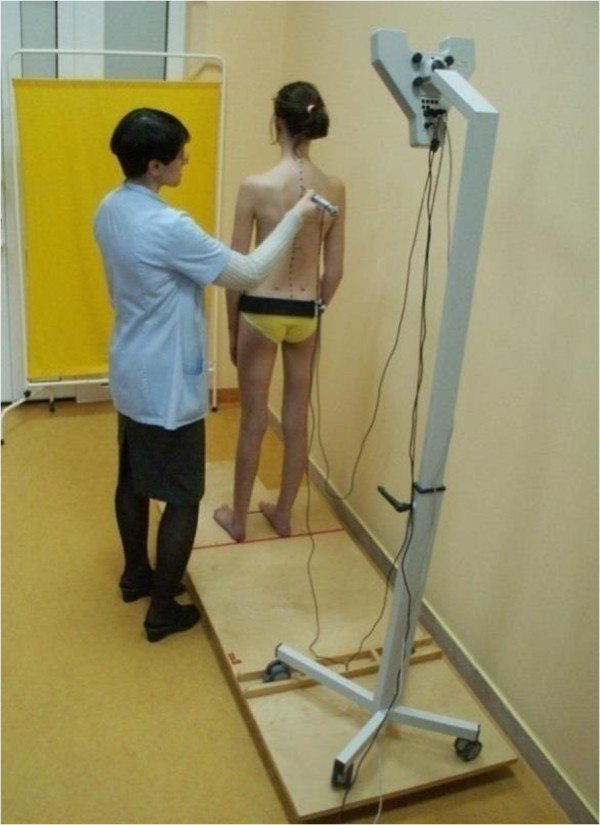
**Examination with Zebris CMS-10.** Ultrasound probe recording the position of marked skeletal landmarks.

Following the clinical evaluation, the same postural parameters were assessed with a Zebris CMS-10 device (Zebris Medical GmbH). The Zebris CMS 10 uses WinSpine software in the Microsoft Windows XP environment. Measurement error is defined by the manufacturer at 1.96 degrees and 2.2 millimeters for all parameters. Measurement sensitivity is 0.2 millimeters and 0.5 degrees. The software includes a data base of projects, patients and individual measurements. The core component of the testing system is a measuring device, an ultrasound point indicator probe and a reference marker [19]. The testing device is placed on an adjustable-height arm. The point indicator probe, which is placed directly onto skeletal reference landmarks on the patient’s body, has two ultrasound markers with their central points aligned with the tip of the probe. The skeletal reference landmarks are all thoracic and lumbar spinous processes of the spine. The software precisely calculates the position of the probe. A reference marker in the form of a belt is attached laterally below the posterior superior iliac spines and anterior superior iliac spines so as not to cover the measurement sites. The reference marker is used to eliminate changes of position during the examination. The testing unit was composed of a platform with built-in levels, a Zebris CMS-10 system and a computer. A transverse valve was mounted at one-third of the length of the platform in order to immobilise the Zebris CMS-10 device. Owing to this, the device could be placed in a fixed position and random movements during use were eliminated. A transverse red line was marked permanently at 80 cm from the transverse valve. One side of a 25 × 25 cm square was drawn on this line. The square was contoured with black lines. The lateral sides of the square were used to indicate where the examinees should place their feet in the standing position. The examinees were also instructed to place their feet in front of the red transverse line (Figure 3). The device was calibrated against the ground before each examination.

For statistical analysis of the clinical vs. Zebris-based assessment, physiological spinal curvatures in the sagittal plane were assigned a value of 0 and accentuation or reduction of the curvatures in the sagittal plane below 30° or above 40° for thoracic kyphosis and below 25° or above 35° for lumbar lordosis was assigned a value of 1. A symmetrical position of the pelvis was assigned a value of - 0, and pelvic rotation, a value of 1. Pelvic rotation was assessed manually as a deficit of rotation of the iliac bone relative to the sacral bone on the left and right side of the body. In the frontal plane, symmetry of the acromions and of the pelvis was assigned a value of - 0, and an asymmetry greater than 1 cm in the vertical dimension, a value of 1. The statistical analysis was conducted in Statistica 7 software package, version 10.1 and based on the calculation of means, percentages and the Chi2 test statistics (empirical and expected), and Cramer’s V statistic, which reflects the strength of association of two parameters. The level of significance was set at p < 0.05.

The parents of the children in the study group had provided written consent for their children to participate in the study. All the patients gave their written consent prior to their inclusion in the study.

The study, funded from a scientific grant, was conducted in the years 2011–2014.

## Results

**Figure 4:**
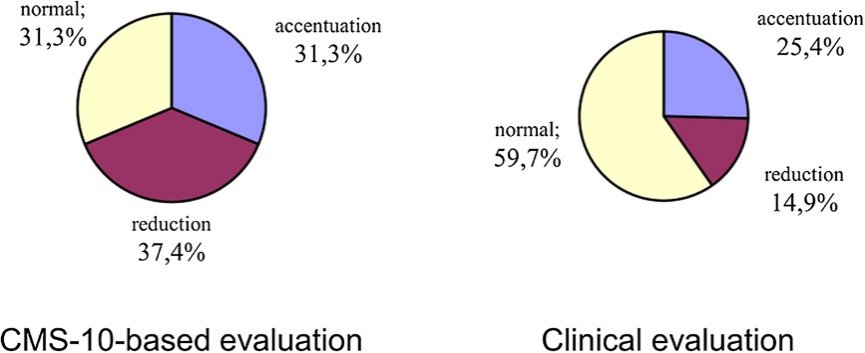
**Results of evaluation of thoracic kyphosis in the sagittal plane in boys.**

**Figure 5:**
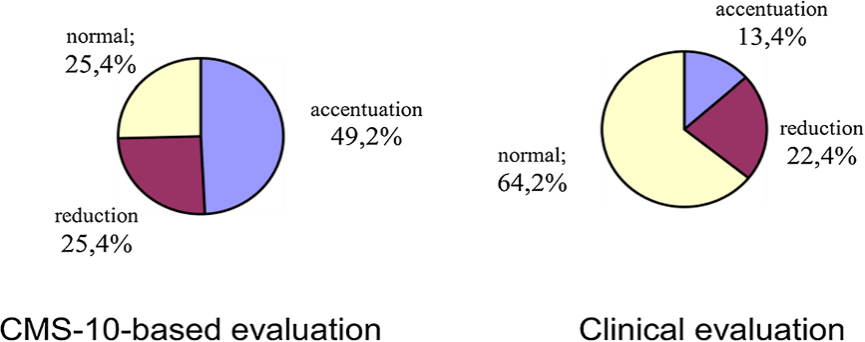
**Results of evaluation of lumbar lordosis in the sagittal plane in boys.**

**Figure 6:**
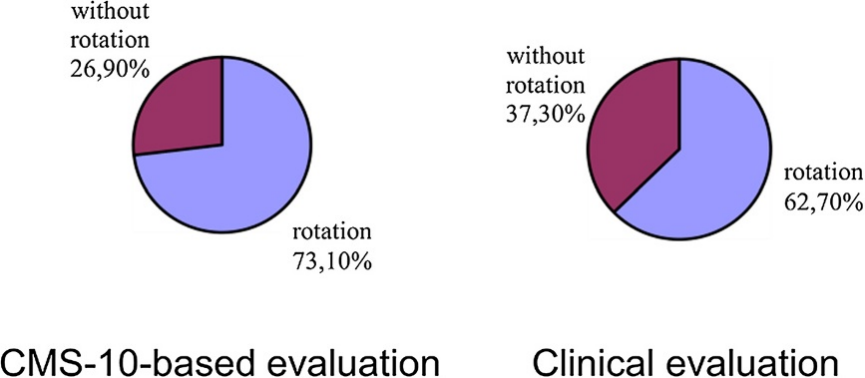
**Results of evaluation of pelvic rotation in the sagittal plane in boys.**

**Figure 7:**
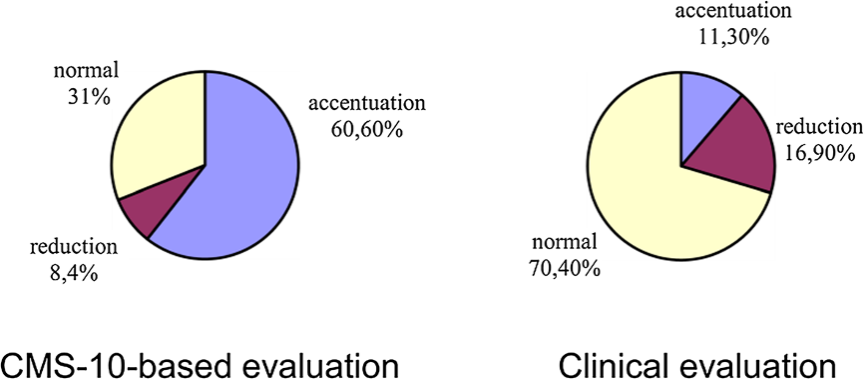
**Results of evaluation of thoracic kyphosis in the sagittal plane in girls.**

**Figure 8:**
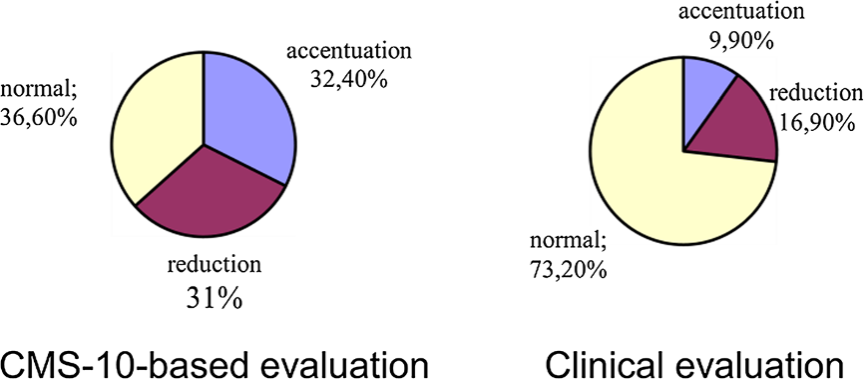
**Results of evaluation of lumbar lordosis in the sagittal plane in girls.**

**Figure 9:**
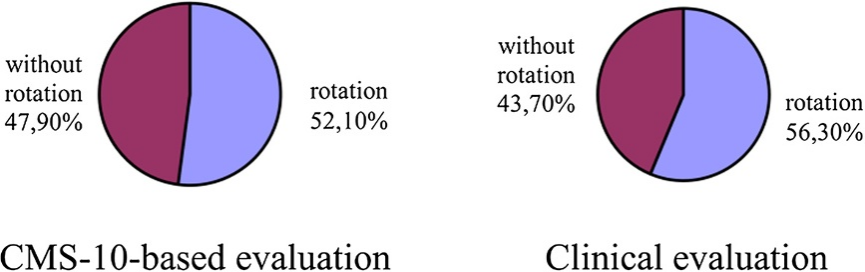
**Results of evaluation of pelvic rotation in the sagittal plane in girls.**

Cramer’s V values confirmed a significant correlation between the parameters in the case of lordosis and a clear correlation in the case of kyphosis. Thus, the study demonstrated that the Zebris CMS-10 system for three-dimensional analysis of body posture contributed a statistically significant adjustment to the clinical evaluation of the spine in the sagittal view; Cramer’s V was 0.514 for the evaluation of thoracic kyphosis in girls and 0.433 in the evaluation of lumbar lordosis in boys (Figures 4, 5, 6, 7, 8, 9).

No statistically significant differences were found with regard to the accuracy of evaluation of pelvic rotation, indicating a similar degree of precision of both techniques, with a Cramer’s V of 0.112 in boys and 0.042 in girls. Similarly, no significant differences were revealed in the frontal plane, also suggesting a similar precision (Tables 1 and 2). These results show that clinical and device-assisted topography is characterised by a similar degree of accuracy. Minor differences were noted with regard to trunk asymmetry in the frontal plane. The differences were not statistically significant (Table 1). Clinical evaluation is thus a reliable method for assessing trunk asymmetry in the frontal plane and does not need to be confirmed by measurement system-based assessment.

**Table 1:** **Trunk assessment in boys, sagittal and frontal planes**

Trunk assessment	CMS -10 evaluation	Clinical evaluation	Calculated Chi ^2^	Significance level p < 0.05	Degrees of freedom	Tabular Chi ^2^	Hypothesis accepted	Cramer’s V
Kyphosis			12.8	0.05	2	5.9	alternative	0.309
Accentuated	21	17						
Reduced	25	10						
Normal	21	40						
Total	67	67						
Lordosis			25.1	0.05	2	5.9	alternative	0.433
Accentuated	33	9						
Reduced	17	15						
Normal	17	43						
Total	67	67						
Pelvis			1.7	0.05	1	3.7	null	0.112
Rotated	49	42						
Not rotated	18	25						
Total	67	67						
Asymmetry			5.88	0.05	2	5.9	null	0.173
Shoulder girdle	48	25						
Inferior scapular angles	45	13						
Pelvic obliqueness	37	28						
Total	130	66

**Table 2:** **Trunk assessment in girls, sagittal and frontal planes**

Trunk assessment	CMS -10 evaluation	Clinical evaluation	Calculated Chi ^2^	Significance level p < 0.05	Degrees of freedom	Tabular Chi ^2^	Hypothesis accepted	Cramer’s V
Kyphosis			37.5	0.05	2	5.9	alternative	0.514
Accentuated	43	8						
Reduced	6	13						
Normal	22	50						
Total	71	71						
Lordosis			20.1	0.05	2	5.9	alternative	0.377
Accentuated	23	7						
Reduced	22	12						
Normal	26	52						
Total	71	71						
Pelvis			0.3	0.05	1	3.7	null	0.042
Rotated	37	40						
Not rotated	34	31						
Total	71	71						
Asymmetry			3.9	0.05	2	5.9	null	0.151
Shoulder girdle	52	24						
Inferior scapular angles	42	18						
Pelvic obliqueness	18	17						
Total	112	59						

## Discussion

Screening tests play a significant role in assessment of normal individual development in the population of children and adolescents. Accurate screening allows for selecting children and adolescents at risk for the development of postural defects or spinal and trunk deformities in order to refer them to appropriate specialists [20]. A clinical examination is the simplest and also the most common form of postural assessment.

In order to make the findings of clinical assessment more objective, measuring devices were gradually introduced in the 20th and 21st centuries, beginning with the Moire method, a photostereometric technique first used by Takasaki in 1970 [21], followed by raster plots projected onto the object being assessed in raster photogrammetry for massive screening tests [22–24]. Modern devices for three-dimensional motion analysis (Metercom system) use the Saunders digital inclinometer and an anthropostereometric technique [15]. Techniques of video capturing of body posture are also available. Importantly, studies comparing postural parameters assessed using different devices do not reveal statistically significant differences in either device-to-device or device-to-clinical examination comparisons [25].

New achievements in objective assessment methods to support clinical examinations based on a mathematical system of three-dimensional body posture analysis were revealed by American and German centres as early as the late 1990’s and in the first decade of this century [26]. German studies show that the Zebris CMS-10 is a precise device that produces a detailed analysis of the trunk position based on anatomical skeletal reference landmarks in static positions with an option to expand sequences of functional movement [27]. Only static positions were analysed in the present study.

The present results confirm that the most difficult aspect of assessment of clinical deformities of the spine is the analysis of pathological spinal curvatures in the sagittal plane, while pelvic rotation and frontal positioning of the trunk are relatively easy to assess clinically. Similar results were obtained by Bibrowicz & Skolimowski [22]. Kyphosis and lordosis are subject to considerable interindividual variability and there are also no standards to define reference ranges for angle values in relation to sex and age in adolescents. The present study confirmed discrepancies between the populations of boys and girls. Abnormal spinal curvatures are one of many problems of adolescence [23]. The development and monitoring of a correct posture during the development of a child and adolescent is a prolonged process that depends on one’s somatic structure and the pace of individual development [3, 8]. The results for the frontal plane showed less discrepancy between the clinical examination and Zebris CMS-10-based assessment. Skolimowski et al. presented similar findings using other research tools [22].

International scientific societies emphasise the need to verify clinical and scientific research to make it more objective. The terminological system proposed by SRS (S*coliosis Research Society)* in 1994 reflects the three-dimensional nature of scoliosis and other spinal deformities [26]. The terminology serves the goal of promoting systematic descriptions of deformities and rationalising and facilitating examinations in clinical practice [28]. Consensus statements published by SOSORT (*Society on Scoliosis Orthopeaedic and Rehabilitation Treatment)* systematise the level of reliability of diagnostic and research procedures employed in the diagnosis of postural defects [2, 10–12].

Our study shows that the Zebris CMS-10 system provides a detailed analysis of the position of set skeletal reference landmarks, thus representing a valuable adjunct to the clinical examination to increase the intrinsic value of screening tests.

## Conclusions

1. The finding of significant discrepancies between the results of assessment in the sagittal plane obtained in the clinical examination and Zebris CMS-10-based assessment suggests that clinical evaluation should be used to provide a general estimation of accentuation or reduction of spinal curvatures in the sagittal plane.
2. The clinical evaluation of posture is reliable with regard to assessment in the frontal plane.
3. The Zebris CMS-10 system makes the clinical examination significantly more objective with regard to assessment of the physiological curvatures and may be used to make screening tests more objective with regard to detecting postural defects.

## Electronic supplementary material

Additional file 1: **Sample examination protocol.** (DOCX 14 KB)
