# Supplementary material for: Objective parallel-forms reliability assessment of 3 dimension real time body posture screening tests
Source: BMC Pediatr. 2014 Sep 4;14:221. doi: 10.1186/1471-2431-14-221 (PMC4169808; doi:10.1186/1471-2431-14-221)
Supplement: Supplementary file 1 — Additional file 1: Sample examination protocol. (DOCX 14 KB) [file 12887_2014_1153_MOESM1_ESM.docx]

|  | | | | SAGITTAL PLANE | | | | | | | | FRONTAL PLANE | | |
| --- | --- | --- | --- | --- | --- | --- | --- | --- | --- | --- | --- | --- | --- | --- |
| NO. | SURNAME AND FIRST NAME | AGE | SEX | HYPHOSIS | | | LORDOSIS | | | PELVIS | | SHOULDER GIRDLE | INFERIOR SCAPULAR ANGLES | PELVIC OBLIQUENESS |
|  |  |  |  | P | S | N | P | S | N | ROTATED | NOT ROTATED |  |  |  |
|  |  |  |  |  |  |  |  |  |  |  |  |  |  |  |
|  |  |  |  |  |  |  |  |  |  |  |  |  |  |  |
